# Cardiac device to plug an aorto-bronchial fistula

**DOI:** 10.1186/s42155-022-00327-w

**Published:** 2022-09-17

**Authors:** Somaye Ahmadi, Parham Sadeghipour, Bahram Mohebbi, Parham Rabiei, Maryam Parham, Hamid Reza Rahmanpour, Jamal Moosavi

**Affiliations:** Cardiovascular Intervention Research Center, Rajaei Cardiovascular Medical and Research Center, Iran University of Medical Sciences, Tehran, Iran

**Keywords:** Dacron tube graft, Coarctoplasty, Post-coarctoplasty pseudoaneurysm, Hemoptysis, Aortobronchial fistula

## Abstract

**Background:**

Post-coarctoplasty aortic pseudoaneurysms constitute a lethal problem occurring in up to 38% of patients with a history of aortic coarctation surgical repair. Such pseudoaneurysms are prone to rupture if managed conservatively and high mortality and morbidity if treated with open surgery. Therefore, the endovascular approach has been proposed for their management.

**Case report:**

We describe a patient with a post-coarctoplasty aortic pseudoaneurysm complicated by an aortobronchial fistula. The case was treated via the endovascular approach (thoracic endovascular aortic repair and endovascular coarctoplasty) with an atrial septal defect occluder device.

**Conclusions:**

Endovascular repair is a feasible, safe, and promising treatment for thoracic aortic pseudoaneurysms secondary to coarctation repair.

## Background

Coarctation is one of the causes of aortic aneurysm formation as a result of a more flexible vessel wall, even after repair (Preventza et al. 2013). Aortic coarctation repair has been based on surgical methods from the outset, although catheter intervention techniques are progressively improving (Preventza et al. 2013). Different surgical approaches are available for coarctation repair, including resection with end-to-end anastomosis, transverse suture repair, patch-graft aortoplasty, subclavian flap aortoplasty, and resection with end-to-end conduit interposition. Despite a successful repair, however, there is a likelihood of long-term complications such as aortic coarctation recurrence, aortic aneurysms, and pseudoaneurysms, which are at risk for dissection, rupture, or fistulization to adjacent structures (Oliver et al. 2004; Moosavi et al. 2022). We herein describe a patient with an aortic pseudoaneurysm at the site of a Dacron tube graft used for coarctoplasty, resulting in an aortobronchial fistula.

## Main text

### Case report

A 43-year-old man presented to our hospital due to massive hemoptysis. He had a history of surgical repair of interrupted aorta via bypass grafting 19 years previously. The patient stated that on the day before his referral, he had suffered dyspnea and pleuritic chest pain, which were subsequently relieved.

At presentation, the patient had a blood pressure of 138/74 mm Hg, a heart rate of 98 beats per minute, a respiratory rate of 16 breaths per minute, and an oxygen saturation level of 97% in room air. He had no fever. Cardiac auscultation revealed normal regular heart sounds. Additionally, the lungs in auscultation were clear, and no crackles or any other abnormal sounds were heard. Other organs were also normal on physical examination.

The patient’s laboratory results are summarized in Table 1.

**Table 1:** Laboratory results

Laboratory parameter	**Result**
Hemoglobin	14.7 g/dl
White blood cell	8100 cells/mm^3^
platelet	234,000 /mm^3^
Erythrocyte sedimentation rate (ESR)	5 mm/hour
Prothrombin time	13.2 s
International normalized ratio	1
Partial Thromboplastin Time	27 s
Creatinine	1 mg/dl (88.42 μmol/L)
**Venous blood gas:**	
**PH**	7.41
**PCO2**	36
**HCO3**	23
**Urine analysis**	normal

**Fig. 1:**
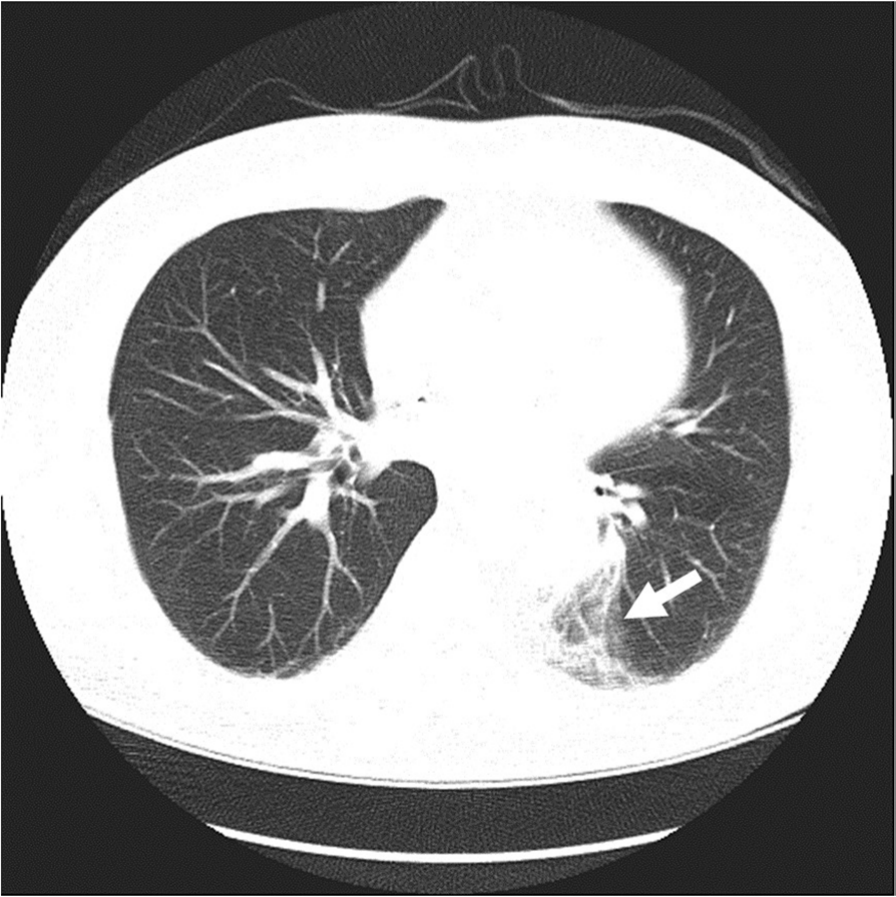
Spiral chest CT depicted a consolidation in posterior aspect of left lung

Spiral chest computed tomography (CT) depicted a consolidation in the posterior aspect of the left lung, just adjacent to the tube graft, in favor of hemorrhage (Fig. 1). Spiral CT angiography of the pulmonary arteries showed no evidence of pulmonary thromboembolism.

**Fig. 2:**
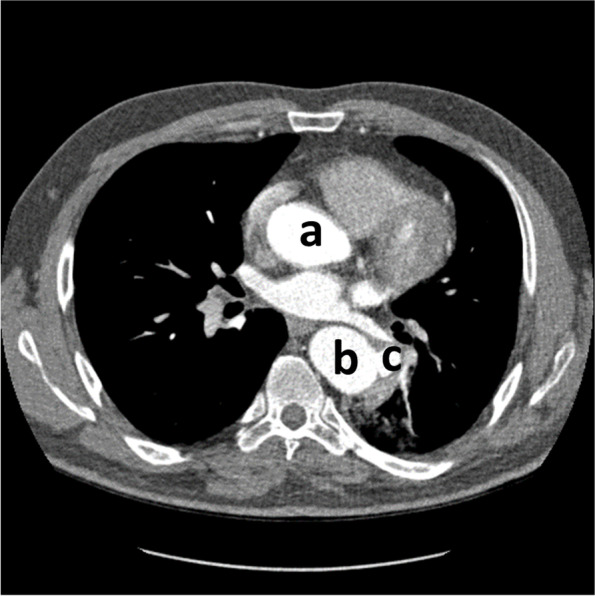
Spiral CT angiography of the Thorasic Aorta revealed two outpouchings from tube graft and fistula to bronchial artery. a: ascending aorta, b: distal anastomosis site of tube graft to descending aorta, c: outpouching from distal anastomosis site of tube graft

**Fig. 3:**
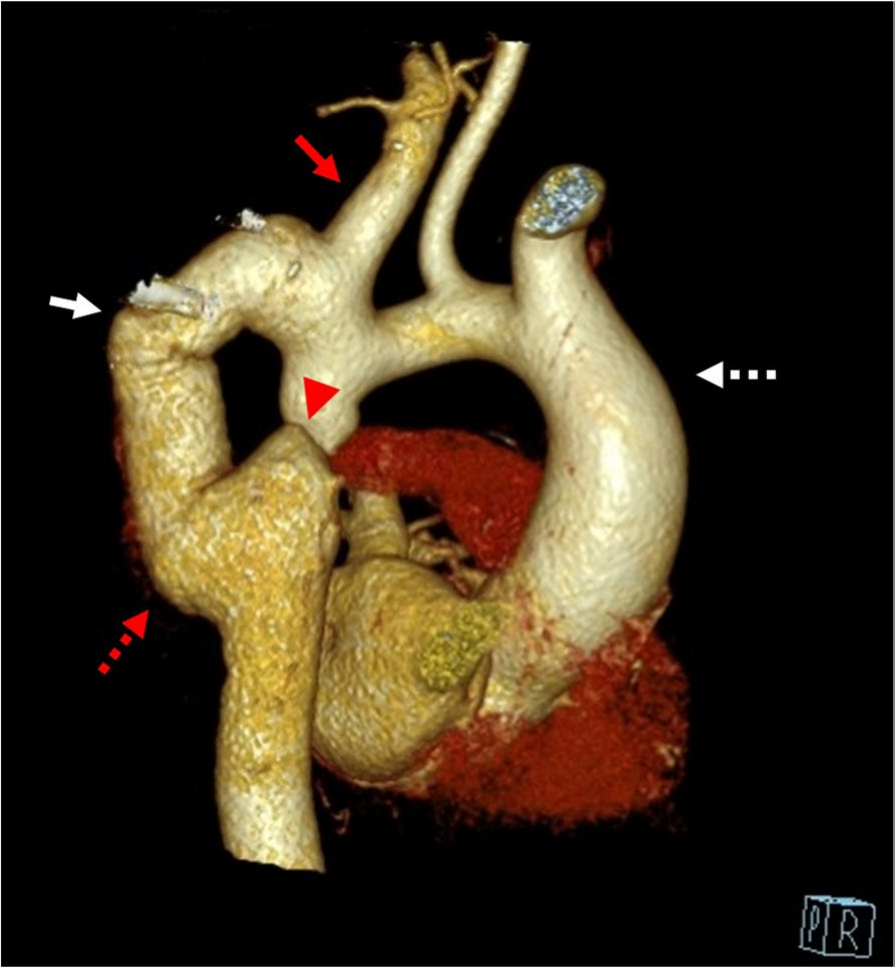
CT angiography of thorasic aorta (3D reconstruction). Red Arrow:the left Subclavian artery is ectatic, reflecting a more diffuse arteriopathy, Arrow head: Aortic interruption, Red Dashed arrow: pseudoaneurysm of distal anastomotic site, White arrow: pseudoaneurysm of mid posterior part of the tube graft. White Dashed Arrow: Ascending Aorta

Spiral CT angiography of the aorta revealed aortic interruption after left subclavian artery debranching and a patent tube graft with its proximal anastomosis onto the left subclavian artery origin and the aorta immediately distal to the left subclavian artery with 2 pseudoaneurysms of about 20 mm: one emanating from the mid-posterior part of the tube graft and the other from the distal anastomotic point with wall thrombosis fistulating into the adjacent pulmonary parenchyma (Figs. 2 & 3). As well, it showed ectatic left Subclavian artery, reflecting a more diffuse arteriopathy.

Accordingly, the patient underwent catheterization.

### Technique

In the catheterization laboratory, arterial access was obtained via the right femoral and radial arteries. Contrast injection in the proximal end of the graft showed that the proximal anastomosis was attached to the ostium of the left subclavian artery. Hence, a sole thoracic endovascular aortic repair (TEVAR) procedure would entail a high risk for endoleaks from the left subclavian artery (type II endoleak).

**Fig. 4:**
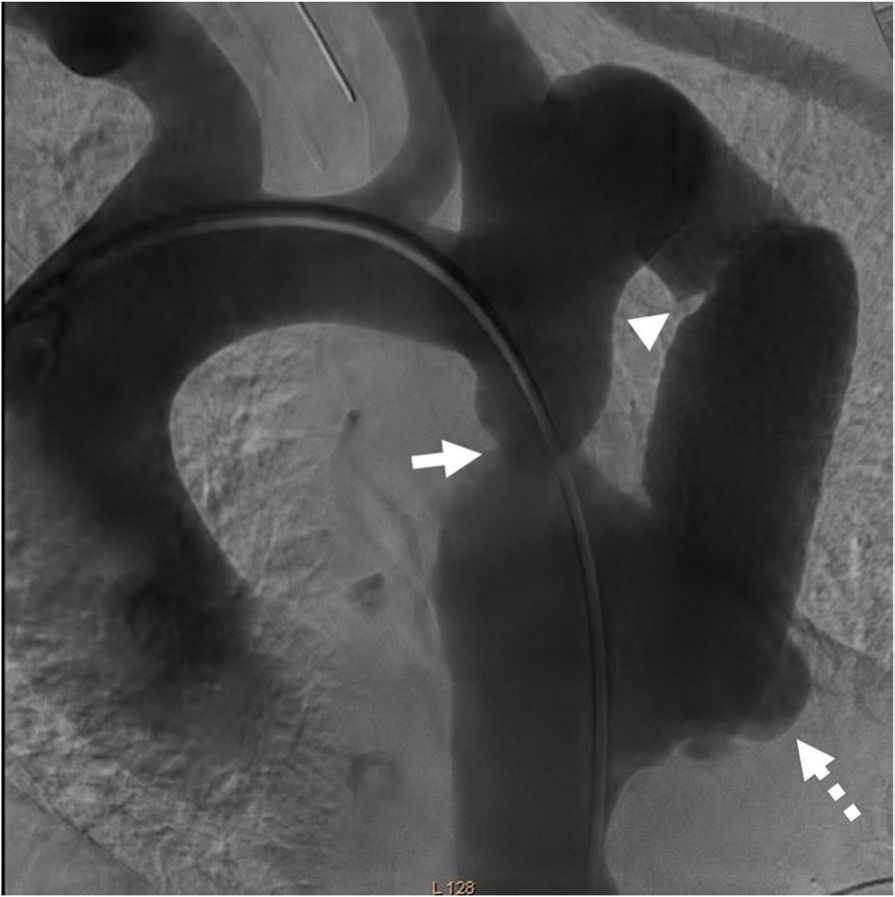
Aortic root injection during fluorosocpy. Confirmed CT angiography results and depicted that proximal anastomotic site emanated from ostioproximal of left subclavian artery. arrowhead: tube graft, arrow: interruption site, dashed arrow: pseudoaneurysm of distal anastomotic site

A Judkins right (JR) catheter was advanced on a 0.035-inch J-tipped guidewire via right radial access to the ascending aorta. Next, the wire was exchanged with a 0.014 BMW guidewire, which was passed through the interruption site. Via right femoral artery access, the BMW was snared with the aid of a 6 F JR catheter and a snare device, and the JR catheter was pulled out. After that, a pigtail catheter was advanced through this wire to the ascending aorta. The tip of the catheter was kept there in order for the contrast material to be injected through it with a view to defining the interruption site and other structures such as anastomotic sites (Fig. 4). The catheter was, then, connected to the pressure system.

In the next step, via left femoral artery access the JR catheter was advanced with a 0.035-inch J-tipped guidewire to the ascending aorta via the tube graft. Then, removed, and was exchanged with a 14 F long sheath (45 cm) using a super-stiff wire. The distal tip of the sheath was left in the proximal portion of the left subclavian artery, and its dilator and the super-stiff guidewire were removed.

In the next stage, for the prevention of retrograde perfusion into the fistula or the pseudoaneurysm site and also for the prevention of endoleaks from the proximal anastomotic site, a Figulla Flex II atrial septal defect occluder device (30 mm, Occlutech) was inserted into the tube graft. Thus, the device, as well as its delivery cable and loader, was prepared, and the loader was attached to the delivery sheath. Next, the device was advanced carefully until it reached the tip of the delivery sheath in the ostium of the left subclavian artery. Under fluoroscopic guidance, the sheath was retracted over the delivery cable, and the left atrial disk was deployed in the proximal portions of the left subclavian artery and the tube graft to prevent endoleaks and exclusion of the fistula and the pseudoaneurysms. Subsequently, with tension on the delivery cable, the sheath was retracted further to deploy the right atrial disk. Before the release of the device, appropriate position and flow limitation were confirmed through contrast injection via the pigtail catheter.

**Fig. 5:**
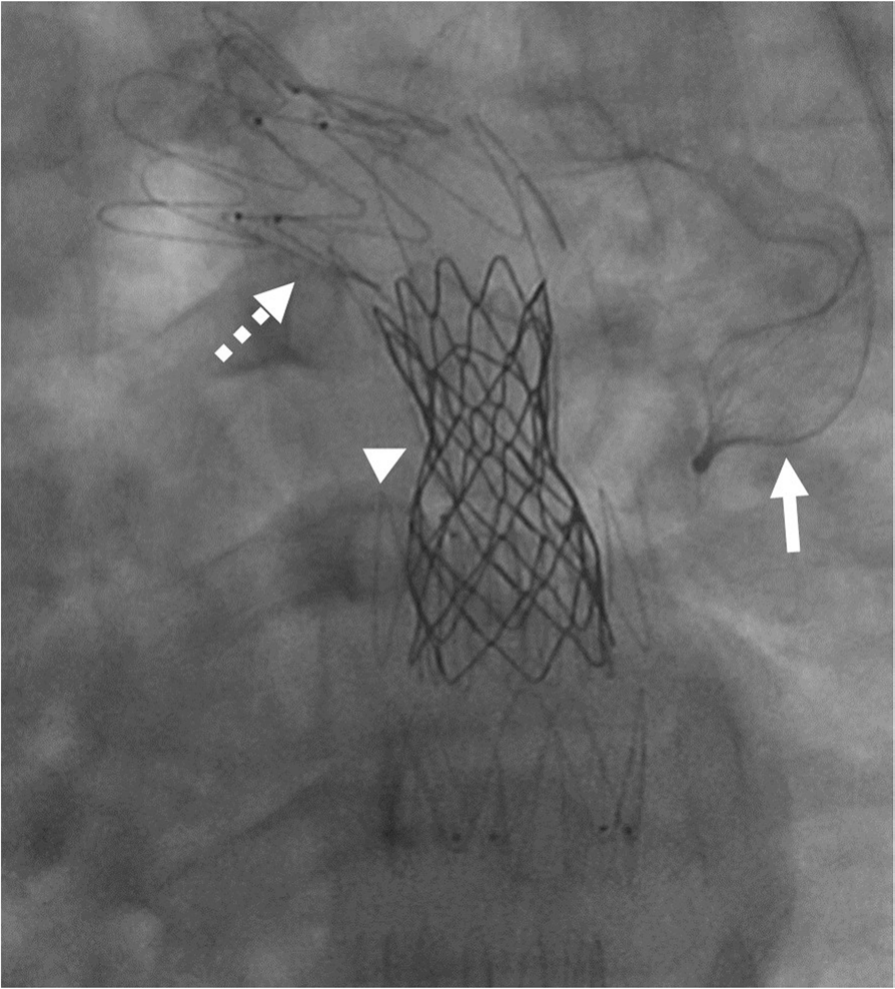
Fluoroscopy after procedure. In order of embedding: Arrow: Occlutech device, dashed arrow: ZENITH stent (for exclusion of tube graft), arrow head: CP stent for coarctoplasty

In the following step, a super-stiff wire was advanced into the pigtail catheter, and its tip was fixed in the ascending aorta. The pigtail catheter was, then, extracted. Afterward, the coarctation site was predilated with a Powerflex Pro Balloon (10 mm × 2 cm) and an Oceanus 35 Balloon (6 mm × 40 mm). TEVAR was performed with an endovascular graft (28–80) (ESBE, Cook Medical) and an endovascular graft (26–10) (Zenith Alpha Thoracic Endovascular Stent Graft, Cook Medical) to exclude the proximal and distal anastomotic sites of the graft. After that, a 4.5 cm CP stent (NuMED Inc, Hopkinton, New York), which was premounted on a BIB balloon (5 cm × 20 cm), was implanted in the aortic interruption site (Fig. 5). The final angiogram showed complete exclusion of the tube graft without any endoleaks.

CP stent insertion into the graft stent eliminated the residual 15 mmHg pressure gradient across the lesion after TEVAR.

### Follow up

**Fig. 6:**
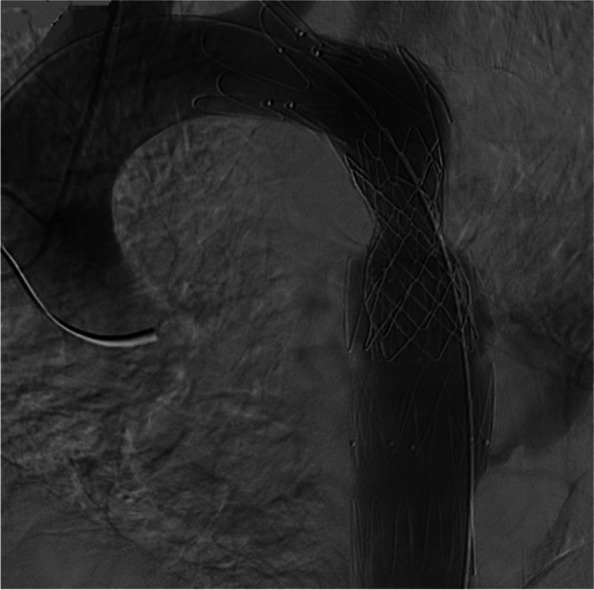
Final aortic root injection revealed effective exclusion of tube graft without endoleak

**Fig. 7:**
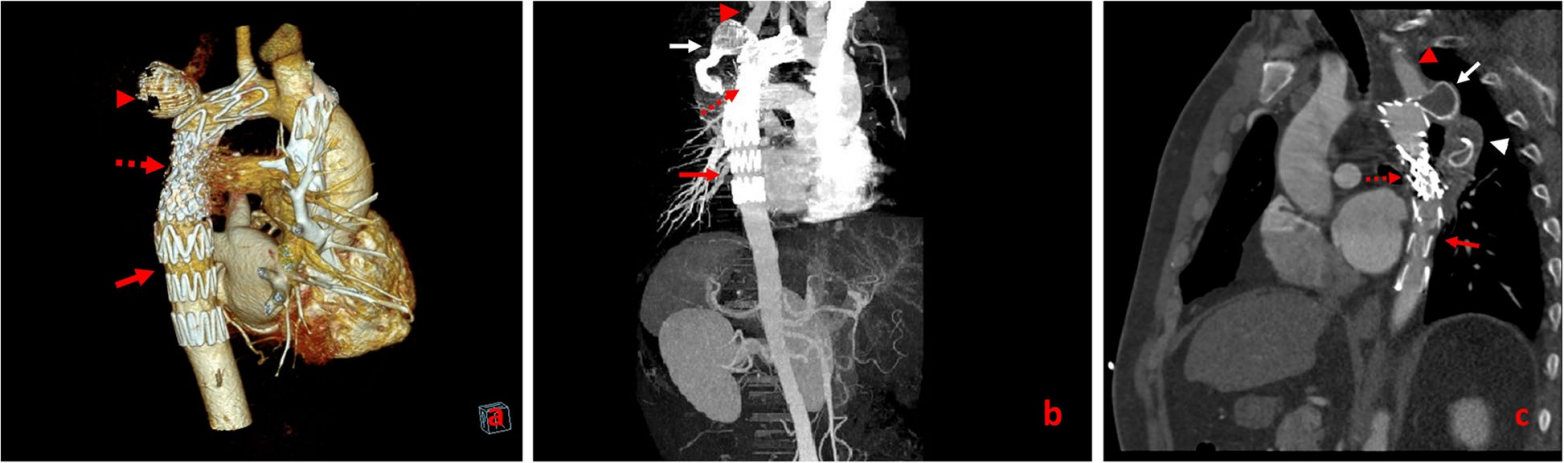
Follow up CTangiography the day after (**a**), one month later (**b**) and after one year (**c**) showed complete exclusion of tube graft without endoleak and successful endovascular coarctoplasty. Red arrow: TEVAR device, dashed arrow: CP stent, Red arrow head: Left Subclavian artery, White arrow: Occlutechdevice, White arrow head: thrombosed excluded tube graft

After the procedure, the patient’s hemoptysis was eradicated. At 1 year’s follow-up, he was in good condition and had experienced no recurrence of symptoms. Aortic CT angiography, conducted at 1 month and 1 year’s follow-ups, revealed complete exclusion of the tube graft without endoleaks and a patent CP stent (Figs. 6 & 7).

In spite of fistula formation, the patient follow up and more evaluation did not unmask any sign related to graft infection.

## Discussion

Surgical treatment of aortic coarctation has a high success rate. However, irrespective of the surgical technique used, a significant portion of patients develop late complications, including re-coarctation, aneurysms, pseudoaneurysms, systemic hypertension, infections including mycotic aneurysm, premature coronary artery disease, aortic valve abnormalities, dissection, and aortic rupture (Oliver et al. 2004). Aneurysms, true or false, are the most common complications, with an incidence rate of 7% to 38%. Asymptomatic enlargement of these kind of aneurysms or pseudoaneurysms has an unacceptable high rate of sudden rupture according to literature even in smaller sizes (Oliver et al. 2004; García-Pavíaa 2010; Kodolitsch 2002). Aneurysms occur following all types of surgical and even transcatheter repair procedures, especially after Dacron patch graft aortoplasty.

Pseudoaneurysms, with an incidence rate of 3% to 38%, develop from suture lines or at the site of isthmic restenosis. Conservative management is associated with an unbelievably high rupture rate and a rupture-related mortality rate of 7% (Oliver et al. 2004; García-Pavíaa 2010; Kodolitsch 2002; Marcheix et al. 2007).

Aneurysm pathogenesis can be explained by congenital weakness (thinning or cystic medial necrosis) of the aortic wall, foreign-body reaction, acquired atherosclerotic changes in the aortic wall, hemodynamic changes (hypertension or turbulence), excessive wall stress originating from the rigid patch onto the more elastic wall opposite to the patch, intimal damage, excessive excision of the coarctation, infection, aortic wall necrosis, and suture fracture (Oliver et al. 2004).

Other risk factors for aneurysm formation include the type of graft (knitted Dacron interposition grafts compared with woven Dacron interposition grafts), bicuspid aortic valves, advanced age at primary coarctation repair, hypoplastic transverse aortic arches, and high preoperative systolic peak pressure gradients (Oliver et al. 2004).

Conventional management of large thoracic aneurysms after aortic coarctation repair is similar to surgical treatment of nonspecific aneurysms. It is a complex procedure in that it necessitates hypothermic circulatory arrest more frequently and is associated with high mortality (14%– 23%) and morbidity, including paralysis of the recurrent laryngeal and phrenic nerves, bleeding, and paraplegia (García-Pavíaa 2010; Yazar et al. 2011). The endovascular approach has been proposed as a promising alternative for managing these patients (García-Pavíaa 2010).

Bertrand Marcheix (2007) described 4 patients with a history of surgical repair of congenital aortic coarctation who suffered from pseudoaneurysms. There were 2 graft interpositions:1 subclavian flap aortoplasty and 1 aorto-aortic bypass. All the patients were treated via the endovascular approach with a mean interval of 24 years from the surgery. One of the patients had massive hemoptysis resulting from an aortobronchial fistula and, thus, received emergent treatment. In all the cases, the Zenith TX2 thoracic stent-graft was used, and 1 patient underwent predilation at the coarctation site. Moreover, no major complications occurred during the procedure, and there was no mortality during the follow-up. One patient presented with a type II endoleak, which spontaneously healed during the first month. Another patient presented with claudication of the left arm resulting from coverage of the left subclavian artery and underwent carotid-subclavian bypass. After a median follow-up of 7.5 months, the patients were asymptomatic, and CT scans revealed complete exclusion of all the aneurysms without any stent-graft-related complications**.**

Omid Shafe (2018) described a 60-year-old man with a history of surgical repair of aortic coarctation who presented with inferior ST-segment-elevation myocardial infarction and simultaneous massive hemoptysis. CT angiography of the thoracic aorta revealed a large and ruptured pseudoaneurysm adjacent to the graft insertion site between the left subclavian artery and the descending thoracic aorta. After primary angioplasty on the occluded right coronary artery via right radial access, the patient underwent exclusion of the ruptured pseudoaneurysm and the graft itself with the aid of a covered CP stent. Thereafter, stent coarctoplasty was performed on the native site of the aortic coarctation using a long self-expandable bare-metal stent. Finally, an occluder was inserted into the previous graft. The final angiogram, as well as follow-up CT angiography, showed no endoleaks.

TEVAR is not free of morbidity and is associated with such complications as endoleaks, neurological and vascular dissection, pneumonia, upper limb claudication, and neurological complications of extracranial vessel rerouting. Still, the design of the newer generations of stents-grafts has conferred more ease of use and durability. Further, redo surgery is associated with complications, and patients tend to opt for minimally invasive procedures. Endovascular stent grafting is considered as the preffered option for the treatment of patients with post-coarctoplasty aortic pseudoaneurysm (García-Pavíaa 2010; Yazar et al. 2011).

## Conclusions

Endovascular repair, given its more feasibility and less invasiveness, is a promising alternative to redo surgery for thoracic aortic pseudoaneurysms secondary to coarctation repair. Nevertheless, clinical and imaging long-term follow-ups are essential to assess the durability of stent-graft repair and to detect possible long-term complications, particularly endoleaks.

## Data Availability

The authors confirm that the data supporting the findings of this study are available within the article.

## Abbreviations

CT: Computed tomography
TEVAR: Thoracic endovascular aortic repair
JR: Judkins right
