# Correction: Cardiac device to plug an aorto-bronchial fistula

**DOI:** 10.1186/s42155-022-00346-7

**Published:** 2022-12-29

**Authors:** Somaye Ahmadi, Parham Sadeghipour, Bahram Mohebbi, Parham Rabiei, Maryam Parham, Hamid Reza Rahmanpour, Jamal Moosavi

**Affiliations:** Cardiovascular Intervention Research Center, Rajaei Cardiovascular Medical and Research Center, Iran University of Medical Sciences, Tehran, Iran

**Correction: CVIR Endovasc 5, 49 (2022)**

**https://doi.org/10.1186/s42155-022-00327-w**

In the originally published version of this article (Ahmadi et al., 2022), the name of the patient was mistakenly included in the consent form. The original article has been corrected.
